# Clinical Implications of Malnutrition in the Management of Patients with Pancreatic Cancer: Introducing the Concept of the Nutritional Oncology Board

**DOI:** 10.3390/nu13103522

**Published:** 2021-10-07

**Authors:** Giulia Rovesti, Filippo Valoriani, Margherita Rimini, Camilla Bardasi, Roberto Ballarin, Fabrizio Di Benedetto, Renata Menozzi, Massimo Dominici, Andrea Spallanzani

**Affiliations:** 1 Division of Oncology, Department of Medical and Surgical Sciences of Children and Adults, University Hospital of Modena and Reggio Emilia, Largo del Pozzo 71, 41125 Modena, Italy; margherita.rimini@gmail.com (M.R.); camilla.bardasi@gmail.com (C.B.); mdominici@unimore.it (M.D.); 2 Division of Metabolic Diseases and Clinical Nutrition, Department of Specialistic Medicines, University Hospital of Modena and Reggio Emilia, Largo del Pozzo 71, 41125 Modena, Italy; valorianifilippo@gmail.com (F.V.); renata.menozzi@unimore.it (R.M.); 3 Division of Hepato-Pancreato-Biliary Surgery and Liver Transplantation, Department of General Surgery, University Hospital of Modena and Reggio Emilia, Largo del Pozzo 71, 41125 Modena, Italy; ballarin.roberto@aou.mo.it (R.B.); fabrizio.dibenedetto@unimore.it (F.D.B.)

**Keywords:** pancreatic cancer, cachexia, malnutrition, survival, sarcopenic obesity, PEI, sarcopenia, nutritional status, QoL, multidisciplinary board

## Abstract

Pancreatic cancer represents a very challenging disease, with an increasing incidence and an extremely poor prognosis. Peculiar features of this tumor entity are represented by pancreatic exocrine insufficiency and an early and intense nutritional imbalance, leading to the highly prevalent and multifactorial syndrome known as cancer cachexia. Recently, also the concept of sarcopenic obesity has emerged, making the concept of pancreatic cancer malnutrition even more multifaceted and complex. Overall, these nutritional derangements play a pivotal role in contributing to the dismal course of this malignancy. However, their relevance is often underrated and their assessment is rarely applied in clinical daily practice with relevant negative impact for patients’ outcome in neoadjuvant, surgical, and metastatic settings. The proper detection and management of pancreatic cancer-related malnutrition syndromes are of primary importance and deserve a specific and multidisciplinary (clinical nutrition, oncology, etc.) approach to improve survival, but also the quality of life. In this context, the introduction of a “Nutritional Oncology Board” in routine daily practice, aimed at assessing an early systematic screening of patients and at implementing nutritional support from the time of disease diagnosis onward seems to be the right path to take.

## 1. Introduction

Despite important efforts in developing pioneering targeted therapies, pancreatic cancer (PC) has today the highest mortality rate of all major cancers. In contrast to other gastrointestinal tumors, pancreatic adenocarcinoma has an increasing incidence. Nowadays, it represents the 4th leading cause of cancer death in the World and it is estimated to reach 2nd place by 2030. Survival remains extremely poor: only 8.1% of patients are alive after 5 years from diagnosis and this percentage drops to 3% after 10 years [1].

The poor response to available treatments and the early patient’s performance deterioration complicate the management of this disease: patients show an early alteration of metabolic state with a fast weight loss, causing enhanced chemotherapy (CHT) toxicities [2,3].

In the last few years, recent trials demonstrated that an early dietary support can positively impact survival, in terms of better tolerance and better response to disease treatments but also improved quality of life (QoL) [4,5].

Patients with pancreatic cancer early show a wide range of nutritional alterations resulting in cancer cachexia (CC), a specific form of disease related-malnutrition (DRM) triggered mainly by the tumor-specific inflammatory response.

Traditionally, CC was defined as a multifactorial syndrome characterized by an ongoing loss of skeletal muscle mass (sarcopenia), with or without loss of fat mass, that cannot be fully reversed by conventional nutritional support and that leads to progressive functional impairment [6]. Standard screening tools based on body weight, weight loss, and food intake can be used to identify CC, but a more specific nutritional assessment is mandatory to classify and treat this metabolic dysregulation [7]. The nutritional assessment consists of a more detailed evaluation of nutritional status, considering nutrient balance, body composition and function, and inflammatory activity [8]. Presentation and severity of cancer-cachexia are heterogeneous and, therefore, its definition is currently evolving and, somehow, controversial. The diagnostic criteria for cancer cachexia include an involuntary weight loss greater than 5% of the usual body weight, or weight loss of more than 2% in patients with a baseline body mass index (BMI) minor than 20 kg/m^2^, over six months [6]. Fearon and collaborators identified three sequential clinical stages in the cancer cachexia process: pre-cachexia, cachexia, and refractory cachexia. In the first stage, patients experience several metabolic alterations converting in impaired glucose metabolism and loss of appetite, without a significant weight loss. When a significant weight loss, according to the overmentioned criteria, takes over, cachexia is established. Finally, cachexia is considered clinically refractory in pre-terminal cancer patients who no longer respond to cancer therapies [6].

The efforts to adjust CC or slow it down can improve patient’s outcomes and lead to more intensive therapeutic strategies. However, the lack of widely agreed diagnostic criteria further complicates the clinical approach to CC. Conventionally, the criteria for sarcopenia in clinical oncology are based on specific computerized tomography (CT) measures of muscularity related to a defined risk of mortality: the current standard consists of evaluating the total muscle cross-sectional area in a region at the third lumbar vertebra (L3) that well correlates with the whole body muscle mass [9]. Some series report the link between specific grades of sarcopenia and overall survival (OS) [10]. In any case, cachexia stratification suffers from patients’ sex, age, and race, from primary tumor location, and other variables, thus making its use difficult to apply.

Due to the complexity of characterization, stratification, and treatment of CC, a multidisciplinary approach to DRM is extremely recommended in daily clinical practice.

## 2. Biological Basis of Malnutrition

Cachexia is a multifactorial syndrome involving cancer and host-derived signaling factors predominantly characterized by non-volitional weight loss and an increased catabolic drive that leads to a profound wasting of fat tissue and sarcopenia [11,12,13]. Cancer cachexia affects approximately 50% of all cancer patients, but in pancreatic cancer setting it amounts to 80% [14].

**Figure 1:**
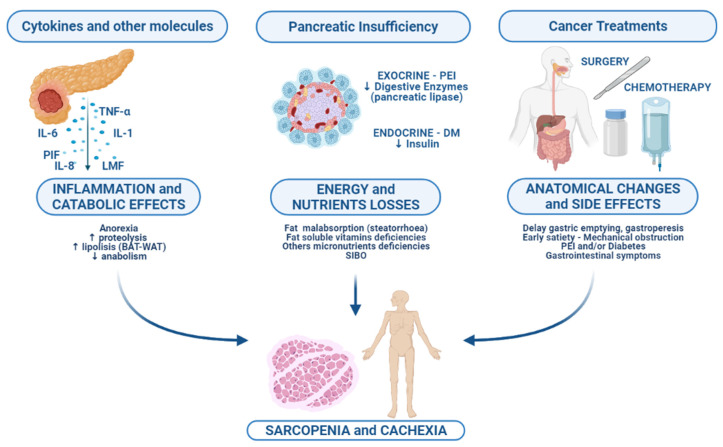
Multifactorial etiology of cachexia and sarcopenia in pancreatic cancer patient. Abbreviations: PIF, proteolysis-inducing factor; LMF, lipid mobilizing factor; BAT, brown adipose tissue; WAT, white adipose tissue; PEI, pancreatic exocrine insufficiency; DM, diabetes mellitus; SIBO, small intestinal bacterial overgrowth.

Since the incidence of cancer cachexia is particularly high in PC patients, it has been proposed that the biological pathways and the host-inflammation response could be more intense in PC compared to other malignancies [15]. The biological mechanisms involved in cancer-associated cachexia are multiple and could be categorized in: catabolic effects derived from the inflammatory state, energy, and nutritional losses, which concern with the pancreatic dysfunction, and anatomical changes due to cancer and side effects of surgical and medical treatments (Figure 1).

### 2.1. Inflammation and Its Catabolic Consequences

The catabolic effects derived by the inflammatory state convert on patient weight loss and sarcopenia and are mediated by several cytokines released from both cancer and host-immune system. In the PC-dependent cachexia, Interleukin-1 (IL-1), IL-6, IL-8, and tumor necrosis factor α (TNF-α) have been recognized as the most involved factors [16]. Particularly, TNF-α has been highlighted as the main pro-cachectic factor involved in lipolysis, proteolysis, insulin-resistance, and muscle atrophy [17]. Along with other cytokines, mostly IL-1 and IL-6, TNF-α acts in combination with the oxidative stress-dependent product nuclear factor-kB (NFkB) by activating the ubiquitin-proteasome pathway, which induces the degradation of regulatory proteins. In particular, once activated NF-kB inhibits the transcription of myoblast determinant protein 1 (MyoD), a myoprotein involved in cell proliferation after muscle damage, inhibiting the ability of myocytes to repair themselves [18]. Moreover, TNF-α is involved in the downregulation of the IGF-1/PI3K/Akt signaling resulting in a reduction in the muscle anabolic capacity. IL-6 cooperates with TNF-α also in stimulating the Janus kinase (JAK)-signal transducer and activator of transcription (STAT), the JAK2/STAT3 pathway, which is involved in inflammation, cancer progression, muscle mass wasting, and cancer-related cachexia [19]. Moreover, TNF-α and IL-6 are both involved in the inhibition of myocytes differentiation, causing an ultrastructural myofiber damage and the replacement of muscle mass with collagen and fat tissue [20]. This mechanism is maintained also by circulating free fatty acids produced through lipolysis, which trigger the secretion of ubiquitin ligases Atrogin-1 and MuRF1 [21].

Chronic inflammation exerts its action by interfering also on several tissues’ function. First of all, the chronic inflammatory state contributes to pancreatic β-cells damage, which could lead to an altered insulin metabolism. The insulin intolerance contributes to the malignant cells’ metabolism, which is based on the recycling of lactate in glucose by the Cory cycle instead of oxidative phosphorylation [22,23]. Secondly, pro-inflammatory cytokines could act directly on the pathways underlying the central anorexia process in PC patients: the Neuropeptide Y (NPY) and the proopiomelanocortin (POMC)/cocaine pathways [24]. Additionally, IL-1 has been shown to upregulate the release of hypothalamic serotonin in experimental models. Serotonin, in turn, contributes to the constitutional activation of POMC/CART (cocaine- and amphetamine-regulated transcript) neurons, resulting in an anorexic effect and in a decrease in appetite [24].

Adipose tissue plays a substantial role in the pathogenesis of cancer cachexia too. Brown adipose tissue (BAT) in adults contains lipid droplets that produce energy by the initiation of a proton leakage pathway in the mitochondrial membrane, under the regulation of the uncoupling protein (UCP) 1 [25]. This process induces the release of reactive oxygen species in skeletal muscle and is associated with the loss of adipose tissue and cancer’s energy imbalance [25]. Moreover, the reduced nutrient intake leads to an increased catabolism of the stored fat, thus contributing to white adipose tissue (WAT) loss and reduction in muscle mass [26].

Cancer itself contributes to the maintenance of metabolic abnormalities by producing several molecules. The lipid mobilizing factor (LMF) has been shown to promote GPT-dependent lipolysis mediated by β3 adrenergic receptors and to contribute to the adipose tissue reaction to other lipolytic stimuli, (e.g., catecholamines) [27]. Another important cancer-derived factor contributing to cancer cachexia is the proteolysis-inducing factor (PIF), which promotes a depletion in muscle mass and in protein synthesis by the activation of the double-stranded RNA-dependent protein kinase (PKR) [28,29,30]. Of note, PIF could activate the ATP-dependent ubiquitin-proteasome pathway (UPP), essential for myofibrillar protein degradation in cancer cachexia [29].

Recently, the new concept of sarcopenic obesity has emerged, resulting in a combination of sarcopenia and obesity, the ‘confluence of two epidemics’ as Roubenoff described [31]. SO is characterized by the highest ranges of fat mass (BMI >30 kg/m^2^) and the lowest ranges of muscularity (high degree of depletion of skeletal muscle mass) and its clinical implications have not been completely defined: the health-related risks may be the same or greater than the respective risks of obesity and sarcopenia alone [31].

Similarly to sarcopenia, obesity is strongly correlated with chronic inflammation and it might impair the immune response to stress [32,33]. The SO resulted to negatively correlate with OS in both resectable and unresectable PC patients [34,35,36,37]; moreover, several evidences demonstrated a correlation between SO and both surgical complications and chemotherapy toxicities [32,34,38]: specific trials are eagerly awaited to clarify whether dose modifications are justified for patients with SO.

### 2.2. Pancreatic Exocrine Insufficiency and Treatment

Pancreatic exocrine insufficiency (PEI) represents another key element involved in pancreatic cancer malnutrition state. It is characterized by the deficiency of pancreatic enzymes that are essential for degradation and consequently for the adsorption of fat, liposoluble vitamins, and antioxidants, resulting in severe maldigestion [39].

The normal pancreatic exocrine activity is characterized by the production of pancreatic juices consisting of digestive enzymes, such as pancreatic amylases and lipases, nucleases, and proteases, such as trypsin, chymotrypsin, elastase, and carboxypeptidase. It is triggered by cholecystokinin (CCK), a hormone secreted by the I-cells of the upper small intestine in response to fat and proteins, binding cholecystokinin A receptor located mainly on pancreatic acinar cells. Simultaneously, the secretin produced by the S-cells of the duodenum stimulates the secretion of HCO3 by pancreatic duct with bile production [40].

PEI can occur in different phases of cancer history: at the onset of the disease, when the primary tumor location is still unknown, after surgery as an early sequela, or during chemotherapy in the advanced stages. It is a multifactorial process: obstruction of the main pancreatic duct, fibrosis of the gland, and loss of pancreatic exocrine tissue are involved [39]. As shown by De la Iglesia et al., PEI has a significantly higher prevalence in tumors located in the head of the pancreas (RR 3.36, 95% CI 1.07–10.54; *p* = 0.04) [41]. Symptoms such as steatorrhea (stool fat content of >7 g/day) associated with abdominal pain, flatulence, and weight loss are typical manifestations; signs of nutrients and liposoluble vitamins deficiency can occur in advanced PEI stage [42,43]. There is no consensus on the diagnosis of PEI. In a recent meta-analysis, different definition of PEI were reported: in four studies it was based on fecal elastase-1, FE-1, (<200 μg/g) [41,44,45,46], in two studies on N-benzoyl-L-tyrosyl-P-aminobenzoic acid (NBTPABA) test [47,48], in one study on triolein or oleic acid [49], in one study on coefficient of fat absorption (CFA) <93% or <85% [50] and in the last study on fecal fat excretion >20 g/d [50]. In this complicated panorama, the degree of PEI measured by FE-1 seems to be strongly associated with poor survival [51]. At the moment of cancer diagnosis, about 50% of patients have laboratory signs of PEI (positive FE-1) but do not present overt symptoms [41].

PEI can be amplified by pancreatic surgery: the alteration of gastrointestinal anatomy with lack of active pancreatic tissue, reduction in postprandial stimulation, and asynchrony between gastric emptying and pancreatic enzyme secretion cause severe maldigestion and complicate malnutrition state. A considerable number of resected cancer patients develop PEI. The severity of post-surgical exocrine insufficiency depends on the underlying disease, preoperative pancreatic function, and extent of the resection [51]. In long term survivors PEI is not the only surgical sequela: the early satiety, the delayed gastric emptying, and gastroparesis lead, when possible, to an extremely slow recovery of dietary status, complicated by pancreatic exocrine and endocrine dysfunctions, with fat and fat-soluble vitamins malabsorption, and the possible development of diabetes mellitus, but also the deficiency of other micronutrients as vitamins B6 and B12 and Fe, Mg, Zn, Cu, and Se [52].

Another PEI-correlated aspect is the possible development of SIBO (“small intestinal bacterial overgrowth”), defined as an increase in the number of bacteria in the small bowel or an alteration in the microorganism’s families. It is extremely common: it occurs in 40%–77% of patients. It is a consequence of bile acid deficiency and it leads to an excessive fermentation and inflammation, thus worsening maldigestion symptomatology. It causes steatorrhea, B12 deficiency, protein-losing, flatulence, abdominal discomfort, bloating, and undernutrition. Furthermore, pancreatic carcinoma’s production of proinflammatory cytokines alters gut barriers with an increase in bacterial translocation [53].

Finally, it should be pointed out that patients with pancreatic carcinoma can also exhibit a pancreatic endocrine insufficiency resulting in diabetes mellitus, termed pancreatogenic diabetes. The ductal obstruction associated with acinar inflammation and fibrosis replacement of the exocrine pancreas seems to predict the development of endocrine pancreas dysfunction [54]. Interestingly, evidence suggests a possible paraneoplastic effect on islet beta-cell function, attested by a bidirectional-blood flow in pancreatic cancer: as described by Chung et al., a continuous beta cell stimulation by acinar and ductal cancer cells (peptide- and cytokine-containing exosomes) produce a high release of insulin and cholecystokinin that promote tumor survival and proliferation [55]. In total, 80% of newly diagnosed patients have abnormal fasting glucose or glucose intolerance; typically, DM is of recent onset (diagnosed less than 24–36 months before the diagnosis of pancreatic cancer) and it seems to be worsened by surgery of primary tumor [56].

The surgical impact on endocrine function is unclear: retrospective studies suggest that the resection of 25–44% of the pancreatic volume during duodenum-pancreatectomy can leads to fasting blood glucose impairment [57,58]. Otherwise, recent findings show endocrine activity after surgery can be unpredictable, resulting in a gain or a loss of function as well [57,59,60]. Burkhart et al. have also shown a major rate of post-surgical DM in patients undergone duodenum-pancreatectomy compared to distal pancreatectomy, underling the important anatomical impairment of these technical procedures and their impact on outcome [61]. Patients with a recent diagnosis of DM or glucose intolerance must, therefore, be screened for pancreatic carcinoma due to the high correlation between these two entities: the deterioration in glucose homeostasis, combined with other potential biomarkers, is under clinical investigation to inform early diagnosis [62].

Malnutrition and malabsorption caused by PEI must be corrected to ensure patients a better QoL and prevent major symptoms, such as steatorrhea. The standard treatment consists of the administration of oral pancreatic enzyme replacement therapy (PERT). Capsules should be swallowed during meals, rather than before or after the meal and there is not a standard dose: the initial dosage should be increased until steatorrhea becomes tolerable [63]. Recently, Sikkens et al. demonstrated that in most cases this supplement is underdosed and this is probably due to different nutritional support needed by patients with different stage diseases [45]. This result has been confirmed by Forsmark et al. who also underlined that PERT is not widespread; in their analysis, only 21.9% of patients with pancreatic cancer received a replacement therapy [64].

Findings concerning clinical and survival implications of PERT are scarce and discordant. In the locally advanced setting, the Japan group of Takanawa Hospital firstly demonstrated a gain in body weight and daily total caloric intake in patients treated with PERT, but without a corresponding survival improvement [48]. Similar results were obtained by Woo et al. [46] and Zdenkowski et al. [65] who did not report any nutritional benefit. However, data from a recent population-based study conducted by Roberts et al. in a cohort of 4554 patients showed that PERT significantly impacts QoL and survival (survival time ratio = 2.62, 95% CI 2.27–3.02) [66]. Patients with LAPC undergoing neoadjuvant CHT are particularly susceptible to severe malnutrition because of the synergy between PEI and oncological treatments that can impair the functional reserve with serious effects on subsequent surgery [66]. In the metastatic setting, PERT positively impacts survival in patients treated with chemotherapy. In a retrospective experience, patients benefiting most from PERT were those who experienced a significant weight loss at diagnosis (HR 2.52; 95% CI 1.55–4.11; *p* < 0.001) [67]. Even if data are controversial, the use of PERT is gaining importance; more and more data are emerging on the potential benefit of replacement therapy on body composition and PERT will probably be included in pancreas treatment guidelines as a co-adjuvant of standard chemotherapy in the near future. More prospective controlled studies with proper sample size are needed to extend scientific understanding and to define a standardized approach.

### 2.3. Cancer Anatomic Changes

The extrinsic compression caused by the tumor, or its surgical resection, can cause anatomic alteration, including mechanical obstruction of the gastrointestinal tract, thus leading to pain, dysphagia, gastroparesis, constipation, and lack of nutrients absorption [13]. Moreover, chemotherapy which constitutes the backbone of treatment in PC patients, could determine nausea, anorexia, and vomiting, thus contributing to the weight depletion and sarcopenia [68].

## 3. Impact of Malnutrition on Clinical Outcome

### 3.1. Neoadjuvant and Preoperatory Setting

Surgical resection remains the only chance of cure in PC; however, it represents an option in less than 20% of patients at diagnosis [69]. Neoadjuvant treatment may increase the resectability rate in borderline resectable (BRPC) and locally advanced (LAPC) at the cost of significant effects on body composition. Sarcopenia occurs at the time of diagnosis in at least half of patients with BRPC and LAPC and weight loss and loss of lean body tissue during chemotherapy are associated with poor outcomes [70].

Naumann et al. analyzed the role of sarcopenia and weight loss in a retrospective cohort of patients with LAPC. The incidence of cachexia (defined as weight loss of 5% or more) and sarcopenia were 85% and 68%, respectively. They observed a significant reduction in muscle mass during chemo-radiotherapy (2.7%) without changes in muscle density. Persistent weight loss (HR 1.556; *p* = 0.028) or muscle mass (HR 1.498; *p* = 0.036) were negatively associated with survival, such as CA 19.9 at follow-up and parenteral nutritional support [71].

Cooper et al. evaluated the role of sarcopenia and depletion of skeletal muscle and adipose tissue in 89 patients with resectable pancreatic cancer previously enrolled in a phase II trial providing the efficacy of neoadjuvant CHT and subsequent chemo-radiotherapy in this setting. Half of patients (52%) were sarcopenic at diagnosis. During treatment, the mean values of skeletal muscle mass, subcutaneous adipose tissue, and visceral adipose tissue (VAT) significantly decreased and 54 patients (62%) lost weight without any impact on resection rate. Patients with sarcopenic obesity (BMI > 30 and skeletal muscle cross-sectional area B < 38.9 cm^2^/m^2^ for women and B < 55.4 cm^2^/m^2^ for men) at diagnosis had a lower probability of radical resection although the correlation did not reach statistical significance (70 vs. 48%; *p* = 0.07). Depletion of skeletal muscle mass was associated with shorter disease-free survival (DFS). In univariate analysis, sarcopenic obesity (SO) at diagnosis (20.7 vs. 12.9 months; *p* = 0.04) and progressive loss of visceral adipose during neoadjuvant therapy (HR 0.97; 95% CI 0.95–0.99; *p* = 0.001) were associated with shorter overall survival (OS), although only the completion of all therapy and in particular radical surgery had an impact in multivariate analysis (HR 0.08; 95% CI 0.04–0.18; *p* = 0.001) [72].

Griffin et al. prospectively recording data from 78 BRPC patients undergoing surgical resection in Dublin National Surgical Center for Pancreatic Cancer from 2012 to 2015, confirmed the incidence of sarcopenia in almost half of patients. They evaluated the role of cachexia and sarcopenia at diagnosis, confirming no correlation with resectability or survival (cachexia HR 0.799, *p* = 0.447, sarcopenia HR 0.841, *p* = 0.497). They also evaluated changes in body composition during chemotherapy, confirming a significant depletion of the amount of adipose tissue (intra-muscular adipose tissue from 9.3 cm^2^ to 7.9 cm^2^, *p* = 0.003; visceral adipose tissue from 143.5 cm^2^ to 111.5 cm^2^, *p* < 0.0001, subcutaneous adipose tissue from 191.2 cm^2^ to 158.5 cm^2^ *p* < 0.0001) and of skeletal muscle (from 45.6 cm^2^/m^2^ to 42.3 cm^2^/m^2^; *p* < 0.001). Loss of lean tissue and depletion of fat mass during neoadjuvant CHT were also associated with higher mortality risk (mean fat-free mass loss 2.6 kg, HR 1.1, *p* = 0.003; mean skeletal muscle mass loss 1.5 kg, HR 1.21, *p* = 0.001; mean loss of fat mass 2.8 kg HR 1.09, *p* = 0.004). Furthermore, they highlighted the role of muscle attenuation (MA) by measuring skeletal muscle radio-density: almost half of the enrolled patients had low MA correlating with an increased mortality risk (median OS 19 months vs. 14 months; HR 0.53, *p* = 0.015) [73].

The link between variation in adipose tissue and muscle tissue during neoadjuvant treatment and resection rate was investigated also by Sandini et al. in a prospective analysis of 193 patients with BRPC and LAPC. In the unresected group, the incidence of sarcopenia increased from 36.8% at diagnosis to 52.8% after chemotherapy, while in the radically resected group, sarcopenia decreased from 46.3% to 36.8%. The amount of adipose tissue decreased during CHT: total adipose tissue area (median pre-treatment: 284.0 cm^2^ vs. post-treatment: 250.0 cm^2^; *p* < 0.001) and visceral adipose tissue area (median pre-treatment: 115.2 cm^2^ vs. post-treatment: 97.7 cm^2^; *p* < 0.001). Differently from the previous Irish trial, the total muscle mass increased (median pre-treatment 122.1 cm^2^ vs. post-treatment: 123.7 cm^2^). Patients who underwent surgery experienced an increase of 5.9% in skeletal muscle tissue instead of a decrease of 1.7% of those without radical surgery (*p* < 0.001) [70].

### 3.2. Surgical Setting

Surgical patients are at high risk of being malnourished and malnutrition is a proven risk factor for postoperative complications in major abdominal surgery. The development of nutritional scores to detect high-risk patients during preoperatory setting has occurred during the last years, but only a small proportion of them has been validated through retrospective trials.

The NURIMAS pancreas trial enrolled 369 patients with resectable pancreatic cancer in Heidelberg from August 2014 to July 2015. The trial evaluated the association between the risk of malnutrition estimated through different nutritional scores and major postoperative complications. None of the considered scores (nutritional risk index, nutritional risk screening score, subjective global assessment, malnutrition universal screening tool, mini nutritional assessment, imperial nutritional screening system, nutritional risk classification, ESPEN malnutrition criteria) provided information about major complications’ risk [74].

Differently, CT-assessed sarcopenia indexes provided an adequate risk evaluation. In a recent meta-analysis by Cao et al., the value of skeletal muscle mass index, SMI, (OR 1.36; *p* = 0.0008), PMI (RR 1.35; *p* = 0.0002), MA (RR 1.40; *p* = 0.002), and IMAC (RR 1.63; *p* < 0.0001) were all predictive of postoperative major complications in biliopancreatic surgery [75].

Focusing on pancreatic surgery, Ratnayake et al. performed a meta-analysis including 33 studies (3608 patients) evaluating the predictive value of preoperative sarcopenia for major complications in pancreatic cancer patients. Sarcopenia was not correlated to postoperative complications, instead it was associated with an increased hospital stay (mean difference of 0.73 days, CI 0.06–1.40, *p* = 0.033) [76].

Two Italian experiences highlighted the link between visceral obesity, specifically sarcopenic obesity, and surgical outcome. Pecorelli et al. analyzed the report of 202 patients undergoing pancreatic surgery between January 2010 and September 2014 and emphasized the association between sarcopenic obesity (visceral fat area/total abdominal muscle area > 3.2) and postoperative death (OR 6.76, *p* < 0.001) and between high visceral fat area and risk of pancreatic fistula (OR 4.05; *p* < 0.001) [34]. A retrospective analysis on 124 patients performed by Sandini et al. confirmed the predictive role of SO for the risk of major surgical complications: in the multivariate analysis, the ratio between VFA (visceral fat area) and TAMA (total abdominal muscle area) was the strongest predictor of major complications, with odds ratios of 3.20 (*p* = 0.008) [32].

An appropriate clinical preoperatory nutritional evaluation might be essential to estimate not only the risk of surgical complications but even more to stratify the long-term survival benefit of patients. A validated screening tool such as the nutrition risk screening 2002 (NRS 2002) score emphasized how a preoperatory malnutritional status could impact overall survival [77].

In addition, preoperatory CT-assessed sarcopenia could be considered not only as a predictive tool but also as a prognostic factor in patients undergoing pancreatic surgery. Almost 10 years ago, Peng et al. evaluated 557 PC patients undergoing curative surgery at John Hopkins University. Sarcopenia remained an independent predictor of survival even in multivariate analysis, together with lesion size, grading, vascular invasion, and lymph node metastasis. Sarcopenic patients had a 63% increased risk of death at 3 years (HR 1.63, *p* < 0.001) [78]. Few years later, Okumura et al. emphasized the prognostic role of muscle quality, as well as muscle quantity. Considering 230 resected PC patients at Kyoto University, 64 had a low psoas muscle index (PMI) and 142 had a high intramuscular adipose tissue content (IMAC). Patients with low PMI and high IMAC were older but without any other difference between patients with high or normal PMI and low or normal IMAC [79]. The median OS in patients with low PMI was 17.7 months, less than in those with normal or high PMI (32 months, *p* < 0.001). The median OS in patients with high IMAC was 21.5 months, less than in those with normal or low IMAC (56.5 months, *p* < 0.001). Low muscle quantity (PMI) and low muscle quality (IMAC) were independent negative prognostic factors for OS (HR 1.999, *p* < 0.001; HR 2.527, *p* < 0.001, respectively) and relapse-free survival (HR 1.607, *p* = 0.007; HR 1.640, *p* = 0.004, respectively) [79].

Recently, the prognostic role of sarcopenia in the surgical setting was emphasized in 2 meta-analyses. Bundred et al. analyzed 42 studies and reported the assessment of body composition in 7619 patients. Sarcopenia was associated with relevant peri-operative mortality (OR 2.40, 95% CI 1.19–4.85, *p* < 0.01) and overall survival in univariate (HR 1.95, 95% CI 1.35–2.81, *p* < 0.001) and multivariate analysis (HR 1.78; 95% CI 1.54–2.05) [80]. Mintziras et al. stressed the impact of SO on 2297 patients enrolled in 11 trials: sarcopenia was significantly associated with poorer overall survival (HR 1.49; 95% CI 1.27–1.74, *p* < 0.001) and, in particular, sarcopenic obesity (reported in 0.6% to 25% of patients) had a stronger impact on overall survival (HR 2.01; 95% CI 1.55–2.61, *p* < 0.001) [81].

### 3.3. Metastatic Setting

In metastatic setting, malnutrition is strictly related to systemic inflammatory response, chemotherapy dose intensity, and survival.

In a multicenter retrospective analysis by Klute et al., the malnutrition evaluated by subjective global assessment (SGA) was an independent prognostic factor for overall survival (median OS not reached versus 8.5 months, *p* < 0.0001) due to its impact on CHT dose reduction (estimate—10.3%; *p* = 0.020) [82].

In the impact study, 94 patients with advanced pancreatic cancer were retrospectively evaluated. Independent from BMI (33% of patients were sarcopenic and 35% of patients had an early loss of skeletal muscle mass despite BMI > 25 at diagnosis), only early loss of more than 10% of SMI and visceral adipose tissue area were significantly associated with worse OS in multivariate analysis (HR 2.16, *p* = 0.007; HR 2.98, *p* = 0.004, respectively) [83].

The prognostic role of baseline sarcopenia at the beginning of FOLFIRINOX polychemotherapy was confirmed both in the first and second lines of treatment. Kurita et al. evaluated 82 patients treated with first-line FOLFIRINOX and they showed that median OS of sarcopenia patients and not sarcopenia patients was 11.3 months and 17 months, respectively (HR 2.49, *p* = 0.001). They also observed that the amount of adipose tissue (adipose tissue index, ATI) and sarcopenic obesity (sarcopenic patients with high ATI) predicted severe hematologic toxicity (*p* = 0.022 and *p* = 0.008, respectively), compromising the efficacy of CHT and highlighting the different role of the amount of muscle and adipose tissue intolerance to chemotherapy [84]. The crucial prognostic role of the amount of adipose tissue during first-line CHT was confirmed by Kays et al. in a retrospective review of 53 patients treated at Indiana University Hospital from 2010 to 2015. They evaluated the amount of SMI and VAT on CT scans during treatment and they eventually developed 3 different cachexia phenotypes: muscle and fat wasting (MFW), only fat wasting (FW), and no wasting (NW). They noted that NW patients (19%) had the greatest median overall survival (22.6 months) without any difference between MFW patients (12.2 months) and FW patients (13.0 months). In multivariate analysis, cachexia phenotype and chemotherapy response were independently associated with survival [85]. Additionally, in the second-line setting, SMI at baseline was the strongest independent prognostic factor in predicting poor prognosis for 57 Korean patients treated in an academic tertiary hospital of South Korea (median OS 4.6 months vs. 8.5 months in patients with low SMI vs. high SMI, respectively, HR 0.469, *p* = 0.015) [86].

**Figure 2:**
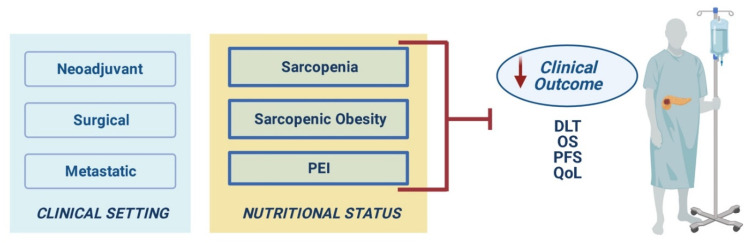
Clinical impact of malnutrition in different treatment settings. Abbreviations: PEI, pancreatic exocrine insufficiency; DLT, dose-limiting toxicities; OS, overall survival; PFS, progression-free survival; QoL, quality of life.

A representative illustration of the clinical impact of malnutrition in different treatment settings is shown in Figure 2.

## 4. From a Solitary Management to a Shared Perspective: The “Nutritional Oncology Board”

Considering the prevalence up to 85% [87], its known and peculiar pathophysiological mechanisms, and the remarkable prognostic relevance, the proper management of pancreatic cancer malnutrition is, undoubtedly, of paramount importance and deserves specific approaches [88].

The European Society for Clinical Nutrition and Metabolism (ESPEN) recently published guidelines on nutritional care in cancer patients and wrote a call-to-action, underscoring its significance and the need for its full translation into practice. The key points of the statement are the following: to screen all cancer patients from the very beginning of the disease course and regularly re-screen, to increase the measures comprised in the nutritional assessment, and to implement nutritional intervention in the form of personalized plans [89].

However, about 50% of malnourished patients still do not receive an adequate nutritional support [90] and this might be due, at least in part, to the fact that the attitude towards the issue of nutritional support for cancer patients is considerably variable among oncologists. Insufficient awareness and the lack of structured teamwork between oncologists and clinical nutritionists are some of the key critical factors that must be fixed to improve cancer care [91]. Moreover, also the need for consistent and harmonized nomenclature and the absence of high-quality randomized clinical trials (RCT)-based evidence of nutritional interventions efficacy are among the obstacles for a diffuse perception and a proper detection of cancer-related malnutrition and cachexia [92]. In a recent paper, Caccialanza and co-authors registered the Italian daily practice in numbers. Regarding the identification of malnutrition, only 16% of the oncology units routinely used validated nutritional screening tools. Percentages were also disappointing regarding the nutritional support, managed by nutrition specialists in just 31% of oncology units. Importantly, when coming to the potential strategies aimed at improving the nutritional care in cancer patients, almost 70% of the oncologists stated that tailored care protocols would be useful to further improve the cancer continuum of care [93]. When assessing a European multinational (Italy, France, Germany) survey regarding the current practices on clinical nutrition intervention, Caccialanza et al. still reported disappointing real-world data, with late and few diagnoses of malnutrition, far distant from what current guidelines recommend [89,94,95], and nutrition interventions often used only in the end-of-life setting, thus losing the possibility of improving patient’s clinical outcome [96].

Following the paper by Caccialanza et al. [93], Rossi R. and coauthors conducted an observational pilot study confirming that only a small proportion of oncologists collects weight parameter, probably considering cancer-associated weight loss unimportant [97], as recently showed also by the European Cancer Patient Coalition study [93]. Importantly, those findings prompted Rossi’s group to build a “nutritional team” to gather oncologists, nutritional experts, and palliative care specialists, giving, therefore, the proof that teamwork is not only the best way to go but probably also the only one [97].

Cancer-related malnutrition causes a cascade of consequences: increased infection rate, increased risk of postoperative complications with prolonged hospitalization, and reduced tolerance or response to CT or radiotherapy lead to increased costs, social burden and reduced performance status, eventually decreasing quality of life [98]. Of note, psychosocial distress can be a not only consequence but also a cause of reduced food intake and malnutrition and might have an impact also on caregivers [99]. Food has both biological and non-biological meanings; eating is a well-recognized social activity with strong psychological implications [100] and diet is one of the very few factors, if not the only one, that patients feel they can somehow control during the entire disease trajectory [101]. When hampered by cancer, eating might lead to emotional distress that is as important as the physical one and that comprises a sense of helplessness and failure, loss of independence, conflictual relationships with caregivers and all family members over food, and isolation. On the other hand, weight loss can be also be experienced as advantageous, especially for overweight and obese patients and at the beginning of the disease course, and this perception might play in nutritional treatment’s disfavor [99].

Considering all of the above, among the results that can be achieved through an integrated approach of care towards cancer malnutrition, there are reduction in post-operative complications, less hospitalization, enhanced likelihood for a patient to be able to face treatments, even the most aggressive and potentially effective ones, and better QoL.

Ten years have passed since Muscaritoli and colleagues suggested the so-called “parallel pathway” as a concept of diagnostic and therapeutic integrated model of intervention, but results, as said, are still disappointing. According to the “parallel pathway” approach, every step in the oncological care is flanked by a metabolic and nutritional intervention: disease staging by nutritional screening and assessment within 4 weeks from diagnosis, elaboration of the oncological treatment plan by nutritional plan, first-line treatment by first-level nutritional intervention, then follow up with periodical re-evaluations until the implementation of a subsequent line of treatment flanked by an upgraded nutritional strategy [102]. Historically, but unfortunately also nowadays, nutritional intervention in cancer has been mainly considered as part of the palliative treatments. Nutritional therapy, instead, must be integrated as early as possible. Timely screening of the nutritional condition has to be considered a hallmark of cancer care and should be carried out through one of the validated tools available [92] and recommended by ESPEN [89]. Patients with screening test positive for nutritional dysfunctions should then be offered a comprehensive assessment in order to detect nutritional intake, symptoms that might hamper it (dysphagia, nausea, vomiting, gastrointestinal problems, psychosocial distress, chronic pain, fatigue, etc.), muscle mass, degree of systemic inflammation, and physical activity [102]. If a deficit in one or more of those tasks is recognized, dieticians and clinical nutritionists, and other specialists if needed, should contribute to remove or at least diminish it [92].

Given the strong benefits for patients, we believe that the introduction of a “Nutritional Oncology Board” (NOB) should be seen as a tool of good clinical practice in nowadays routine care. In order to face malnutrition, the NOB should implement the following interventions: early nutritional assessment before the start of oncological treatment in order to provide a patient-tailored program aimed at preventing or treating sarcopenia/sarcopenic obesity, management of possible endocrine or exocrine insufficiency with the appropriate replacement therapy, carefully and systematically re-evaluation of the patient in order to escalate the nutritional support as soon as needed.

**Figure 3:**
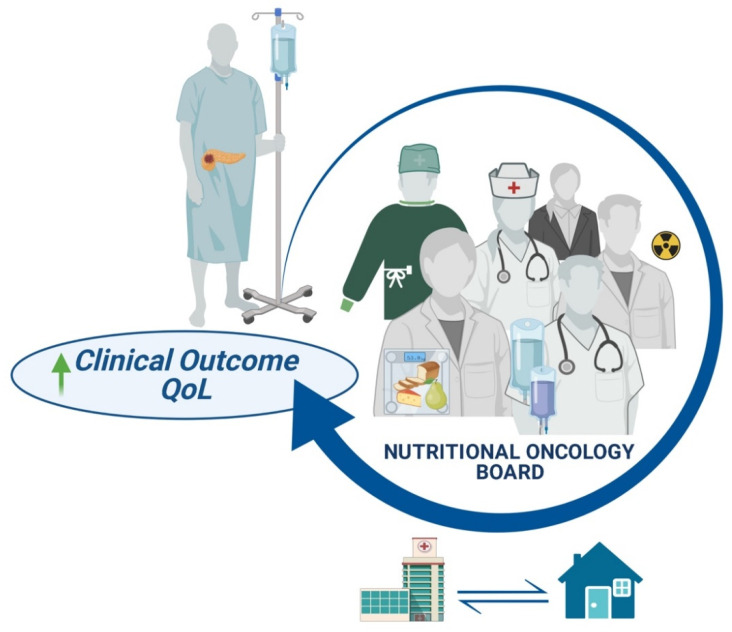
Multidisciplinary management of pancreatic cancer patients. Abbreviations: QoL, quality of life.

Clearly, the adoption of a NOB might require modifications in the daily organization of assistance, but we believe that this effort will be worthwhile. The modality to access the NOB needs to be defined so that activation is systematic and occurs in a fluent way. It is important to define the members of the multidisciplinary team and to figure out who is going to assess the nutritional screening. Considering their specific training and skills, clinical nutritionists and dieticians represent the backbone of the NOB, but successful results might not be obtained without the support of other figures, such as nurses, psychologists, and pain experts, when specific issues are raised during the shared counseling. Operative procedures, such as the so-called “Diagnostic Therapeutic Assistance Path (PDTA)” might represent a useful tool through which a pancreatic cancer patient-centered program of care can be shared by different experts (surgeons, oncologists, radiotherapists, clinical nutritionists, dieticians, etc.) since the very beginning, thus avoiding waste of precious time and allowing a better and forward-looking implementation of nutritional support. The major purpose of nutritional intervention is to maintain pancreatic cancer patients fit for any modality of anticancer therapies, whether it is surgery, radiotherapy, antitumoral drugs, or a combination of them [92]. Clearly, as for many other aspects of supportive care, the integration with the territory is mandatory to ensure a continuous nutritional support, even towards the end-stage of disease when considered appropriate, with the ultimate goal of improving not only patient’s clinical outcome but also the quality of life, reducing anxiety, concern and, more comprehensively, psychological discomfort (Figure 3). Last, but not least, the Nutritional Oncology Board, sharing common experiences, goals, obstacles, and unmet needs can be an optimal fertile ground for the birth of collaborative research activities aimed at enhancing the shared pathway of care from both the clinical and the organizational point of view and, ideally, also at improving the awareness towards this relevant topic.

## References

[1] AIRTUM Working Group I Numeri del Cancro in Italia 2019. Pancreas Esocrino. https://www.aiom.it/wp-content/uploads/2019/09/2019_Numeri_Cancro-operatori-web.pdf.

[2] Tumas J., Tumiene B., Jurkeviciene J., Jasiunas E., Sileikis A. (2020). Nutritional and Immune Impairments and Their Effects on Outcomes in Early Pancreatic Cancer Patients Undergoing Pancreatoduodenectomy. Clin. Nutr..

[3] Kamarajah S.K., Bundred J. (2019). Comments on: Sarcopenia and Sarcopenic Obesity are Significantly Associated with Poorer Overall Survival in Patients with Pancreatic Cancer: Systematic Review and Meta-Analysis. Int. J. Surg..

[4] Richter E., Denecke A., Klapdor S., Klapdor R. (2012). Parenteral Nutrition Support for Patients with Pancreatic Cancer—Improvement of the Nutritional Status and the Therapeutic Outcome. Anticancer Res..

[5] Latenstein A.E.J., Dijksterhuis W.P.M., Mackay T.M., Beijer S., Van Eijck C.H.J., De Hingh I.H.J.T., Molenaar I.Q., Van Oijen M.G.H., Van Santvoort H.C., De Van Der Schueren M.A.E. (2020). Cachexia, Dietetic Consultation, and Survival in Patients with Pancreatic and Periampullary Cancer: A Multicenter Cohort Study. Cancer Med..

[6] Fearon K., Strasser F., Anker S.D., Bosaeus I., Bruera E., Fainsinger R.L., Jatoi A., Loprinzi C., MacDonald N., Mantovani G. (2011). Definition and Classification of Cancer Cachexia: An International Consensus. Lancet Oncol..

[7] Sobotka L. (2004). Basics in Clinical Nutrition.

[8] Soeters P.B., Reijven P.L., Schueren M.A.V.B.-D.V.D., Schols J.M., Halfens R.J., Meijers J.M., van Gemert W.G. (2008). A Rational Approach to Nutritional Assessment. Clin. Nutr..

[9] Shen W., Punyanitya M., Wang Z., Gallagher D., St.-Onge M.-P., Albu J., Heymsfield S.B., Heshka S. (2004). Total Body Skeletal Muscle and Adipose Tissue Volumes: Estimation from a Single Abdominal Cross-Sectional Image. J. Appl. Physiol..

[10] Hilmi M., Jouinot A., Burns R., Pigneur F., Mounier R., Gondin J., Neuzillet C., Goldwasser F. (2019). Body Composition and Sarcopenia: The Next-Generation of Personalized Oncology and Pharmacology?. Pharmacol. Ther..

[11] Porporato P.E. (2016). Understanding Cachexia as a Cancer Metabolism Syndrome. Oncogenesis.

[12] Petruzzelli M., Wagner E.F. (2016). Mechanisms of Metabolic Dysfunction in Cancer-Associated Cachexia. Genes Dev..

[13] Tan C.R., Yaffee P.M., Jamil L.H., Lo S.K., Nissen N., Pandol S.J., Tuli R., Hendifar A.E. (2014). Pancreatic Cancer Cachexia: A Review of Mechanisms and Therapeutics. Front. Physiol..

[14] Aoyagi T., Terracina K.P., Raza A., Matsubara H., Takabe K. (2015). Cancer Cachexia, Mechanism and Treatment. World J. Gastrointest. Oncol..

[15] Gilliland T.M., Villafane-Ferriol N., Shah K.P., Shah R.M., Cao H.S.T., Massarweh N.N., Silberfein E.J., Choi E.A., Hsu C., McElhany A.L. (2017). Nutritional and Metabolic Derangements in Pancreatic Cancer and Pancreatic Resection. Nutrients.

[16] Martignoni M.E., Kunze P., Hildebrandt W., Künzli B., Berberat P., Giese T., Klöters O., Hammer J., Büchler M.W., Giese N.A. (2005). Role of Mononuclear Cells and Inflammatory Cytokines in Pancreatic Cancer-Related Cachexia. Clin. Cancer Res..

[17] O’Riordain M.G., Falconer J.S., Maingay J., Fearon K.C., Ross J.A. (1999). Peripheral Blood Cells from Weight-Losing Cancer Patients Control the Hepatic Acute Phase Response by a Primarily Interleukin-6 Dependent Mechanism. Int. J. Oncol..

[18] Guttridge D.C., Mayo M.W., Madrid L.V., Wang C.-Y., Baldwin A.S. (2000). NF-kappa B-Induced Loss of MyoD Messenger RNA: Possible Role in Muscle Decay and Cachexia. Science.

[19] Yakovenko A., Cameron M., Trevino J.G. (2018). Molecular Therapeutic Strategies Targeting Pancreatic Cancer Induced Cachexia. World J. Gastrointest. Surg..

[20] Judge S.M., Nosacka R.L., Delitto D., Gerber M.H., Cameron M.E., Trevino J.G., Judge A.R. (2018). Skeletal Muscle Fibrosis in Pancreatic Cancer Patients with Respect to Survival. JNCI Cancer Spectr..

[21] Tisdale M.J. (1999). Wasting in Cancer. J. Nutr..

[22] Dev R., Bruera E., Dalal S. (2018). Insulin Resistance and Body Composition in Cancer Patients. Ann. Oncol..

[23] Chen Z., Lu W., Garcia-Prieto C., Huang P. (2007). The Warburg Effect and Its Cancer Therapeutic Implications. J. Bioenerg. Biomembr..

[24] Dunn A.J. (2006). Effects of Cytokines and Infections on Brain Neurochemistry. Clin. Neurosci. Res..

[25] Collins P., Bing C., McCulloch P.B., Williams G.M. (2002). Muscle UCP-3 mRNA Levels are Elevated in Weight Loss Associated with Gastrointestinal Adenocarcinoma in Humans. Br. J. Cancer.

[26] Kandarian S.C., Nosacka R.L., Delitto A.E., Judge A.R., Judge S., Ganey J.D., Moreira M.J., Jackman R.W. (2018). Tumour-Derived Leukaemia Inhibitory Factor is a Major Driver of Cancer Cachexia and Morbidity in C26 Tumour-Bearing Mice. J. Cachexia Sarcopenia Muscle.

[27] Sanders P.M., Tisdale M.J. (2004). Role of Lipid-Mobilising Factor (LMF) in Protecting Tumour Cells from Oxidative Damage. Br. J. Cancer.

[28] Lorite M.J., Thompson M.G., Drake J.L., Carling G., Tisdale M.J. (1998). Mechanism of Muscle Protein Degradation Induced by a Cancer Cachectic Factor. Br. J. Cancer.

[29] Whitehouse A.S., Tisdale M.J. (2003). Increased Expression of the Ubiquitin—Proteasome Pathway in Murine Myotubes by Proteolysis-Inducing Factor (PIF) is Associated with Activation of the Transcription Factor NF-κB. Br. J. Cancer.

[30] Eley H.L., Russell S.T., Baxter J.H., Mukerji P., Tisdale M.J. (2007). Signaling Pathways Initiated by β-hydroxy-β-methylbutyrate to Attenuate the Depression of Protein Synthesis in Skeletal Muscle in Response to Cachectic Stimuli. Am. J. Physiol. Metab..

[31] Roubenoff R. (2008). Excess Baggage: Sarcopenia, Obesity, and Cancer Outcomes. Lancet Oncol..

[32] Sandini M., Bernasconi D.P., Fior D., Molinelli M., Ippolito D., Nespoli L., Caccialanza R., Gianotti L. (2016). A High Visceral Adipose Tissue-to-Skeletal Muscle Ratio as a Determinant of Major Complications after Pancreatoduodenectomy for Cancer. Nutrients.

[33] Nishigori T., Tsunoda S., Okabe H., Tanaka E., Hisamori S., Hosogi H., Shinohara H., Sakai Y. (2016). Impact of Sarcopenic Obesity on Surgical Site Infection after Laparoscopic Total Gastrectomy. Ann. Surg. Oncol..

[34] Pecorelli N., Carrara G., DE Cobelli F., Cristel G., Damascelli A., Balzano G., Beretta L., Braga M. (2016). Effect of Sarcopenia and Visceral Obesity on Mortality and Pancreatic Fistula Following Pancreatic Cancer Surgery. Br. J. Surg..

[35] Tan B.H., Birdsell L.A., Martin L., Baracos V.E., Fearon K.C. (2009). Sarcopenia in an Overweight or Obese Patient Is an Adverse Prognostic Factor in Pancreatic Cancer. Clin. Cancer Res..

[36] Dalal S., Hui D., Bidaut L., Lem K., Del Fabbro E., Crane C., Reyes-Gibby C.C., Bedi D., Bruera E. (2012). Relationships Among Body Mass Index, Longitudinal Body Composition Alterations, and Survival in Patients with Locally Advanced Pancreatic Cancer Receiving Chemoradiation: A Pilot Study. J. Pain Symptom Manag..

[37] Rollins K., Tewari N., Ackner A., Awwad A., Madhusudan S., Macdonald I., Fearon K.C., Lobo D.N. (2016). The Impact of Sarcopenia And Myosteatosis on Outcomes of Unresectable Pancreatic Cancer or Distal Cholangiocarcinoma. Clin. Nutr..

[38] Prado C.M., Lieffers J.R., McCargar L.J., Reiman T., Sawyer M.B., Martin L., Baracos V.E. (2008). Prevalence and Clinical Implications of Sarcopenic Obesity in Patients with Solid Tumours of The Respiratory and Gastrointestinal Tracts: A Population-Based Study. Lancet Oncol..

[39] Vujasinovic M., Valente R., Del Chiaro M., Permert J., Löhr J.-M. (2017). Pancreatic Exocrine Insufficiency in Pancreatic Cancer. Nutrients.

[40] Lindkvist B. (2013). Diagnosis and Treatment of Pancreatic Exocrine Insufficiency. World J. Gastroenterol..

[41] De La Iglesia D., Avci B., Kiriukova M., Panic N., Bozhychko M., Sandru V., De-Madaria E., Capurso G. (2020). Pancreatic Exocrine Insufficiency and Pancreatic Enzyme Replacement Therapy in Patients with Advanced Pancreatic Cancer: A Systematic Review and Meta-Analysis. United Eur. Gastroenterol. J..

[42] Azer S., Sankararaman S. (2021). Steatorrhea.

[43] Halloran C., Cox T., Chauhan S., Raraty M.G., Sutton R., Neoptolemos J., Ghaneh P. (2011). Partial Pancreatic Resection for Pancreatic Malignancy Is Associated with Sustained Pancreatic Exocrine Failure and Reduced Quality of Life: A Prospective Study. Pancreatology.

[44] Partelli S., Frulloni L., Minniti C., Bassi C., Barugola G., D’Onofrio M., Crippa S., Falconi M. (2012). Faecal Elastase-1 is an Independent Predictor of Survival in Advanced Pancreatic Cancer. Dig. Liver Dis..

[45] Sikkens E.C., Cahen D.L., de Wit J., Looman C.W., van Eijck C., Bruno M.J. (2014). A Prospective Assessment of the Natural Course of the Exocrine Pancreatic Function in Patients with a Pancreatic Head Tumor. J. Clin. Gastroenterol..

[46] Woo S.M., Joo J., Kim S.Y., Park S.-J., Han S.-S., Kim T.H., Koh Y.H., Chung S.H., Kim Y.-H., Moon H. (2016). Efficacy of Pancreatic Exocrine Replacement Therapy for Patients with Unresectable Pancreatic Cancer in a Randomized Trial. Pancreatology.

[47] Saito T., Hirano K., Isayama H., Nakai Y., Saito K., Umefune G., Akiyama D., Watanabe T., Takagi K., Hamada T. (2017). The Role of Pancreatic Enzyme Replacement Therapy in Unresectable Pancreatic Cancer. Pancreas.

[48] Saito T., Nakai Y., Isayama H., Hirano K., Ishigaki K., Hakuta R., Takeda T., Saito K., Umefune G., Akiyama D. (2018). A Multicenter Open-Label Randomized Controlled Trial of Pancreatic Enzyme Replacement Therapy in Unresectable Pancreatic Cancer. Pancreas.

[49] Perez M.M., Newcomer A.D., Moertel C.G., Go V.L.W., DiMagno E.P. (1983). Assessment of Weight Loss, Food Intake, Fat Metabolism, Malabsorption, and Treatment of Pancreatic Insufficiency in Pancreatic Cancer. Cancer.

[50] van Nierop J.E.W., Lochtenberg-Potjes C.M., Wierdsma N.J., Scheffer H.J., Kazemier G., Ottens-Oussoren K., Meijerink M.R., de van der Schueren M.A.E. (2017). Assessment of Nutritional Status, Digestion and Absorption, and Quality of Life in Patients with Locally Advanced Pancreatic Cancer. Gastroenterol. Res. Pract..

[51] Pezzilli R., Caccialanza R., Capurso G., Brunetti O., Milella M., Falconi M. (2020). Pancreatic Enzyme Replacement Therapy in Pancreatic Cancer. Cancers.

[52] Petzel M.Q.B., Hoffman L. (2017). Nutrition Implications for Long-Term Survivors of Pancreatic Cancer Surgery. Nutr. Clin. Pract..

[53] Memba R., Duggan S.N., Ni Chonchubhair H.M., Griffin O.M., Bashir Y., O’Connor D.B., Murphy A., McMahon J., Volcov Y., Ryan B. (2017). The Potential Role of Gut Microbiota in Pancreatic Disease: A Systematic Review. Pancreatology.

[54] Rickels M.R., Norris A.W., Hull R.L. (2020). A Tale of Two Pancreases: Exocrine Pathology and Endocrine Dysfunction. Diabetologia.

[55] Chung K.M., Singh J., Lawres L., Dorans K.J., Garcia C., Burkhardt D., Robbins R., Bhutkar A., Cardone R., Zhao X. (2020). Endocrine-Exocrine Signaling Drives Obesity-Associated Pancreatic Ductal Adenocarcinoma. Cell.

[56] Pereira S., Oldfield L., Ney A., Hart P.A., Keane M.G., Pandol S.J., Li D., Greenhalf W., Jeon C.Y., Koay E.J. (2020). Early Detection of Pancreatic Cancer. Lancet Gastroenterol. Hepatol..

[57] Kang J.S., Jang J.-Y., Kang M.J., Kim E., Jung W., Chang J., Shin Y., Han Y., Kim S.-W. (2016). Endocrine Function Impairment after Distal Pancreatectomy: Incidence and Related Factors. World J. Surg..

[58] Shirakawa S., Matsumoto I., Toyama H., Shinzeki M., Ajiki T., Fukumoto T., Ku Y. (2012). Pancreatic Volumetric Assessment as a Predictor of New-Onset Diabetes Following Distal Pancreatectomy. J. Gastrointest. Surg..

[59] Rault A., SaCunha A., Klopfenstein D., Larroudé D., Epoy F.N.D., Collet D., Masson B. (2005). Pancreaticojejunal Anastomosis is Preferable to Pancreaticogastrostomy after Pancreaticoduodenectomy for Longterm Outcomes of Pancreatic Exocrine Function. J. Am. Coll. Surg..

[60] Park J.W., Jang J., Kim E., Kang M.J., Kwon W., Chang Y.R., Han I.W., Kim S. (2013). Effects of Pancreatectomy on Nutritional State, Pancreatic Function and Quality of Life. Br. J. Surg..

[61] Burkhart R.A., Gerber S.M., Tholey R.M., Lamb K.M., Somasundaram A., McIntyre C.A., Fradkin E.C., Ashok A.P., Felte R.F., Mehta J.M. (2015). Incidence and Severity of Pancreatogenic Diabetes after Pancreatic Resection. J. Gastrointest. Surg..

[62] Maitra A., Sharma A., Brand R.E., Eeden S.K.V.D., Fisher W.E., Hart P.A., Hughes S.J., Mather K.J., Pandol S.J., Park W.G. (2018). A Prospective Study to Establish a New-Onset Diabetes Cohort. Pancreas.

[63] Dominguez-Munoz J.E. (2007). Pancreatic Enzyme Therapy for Pancreatic Exocrine Insufficiency. Curr. Gastroenterol. Rep..

[64] Forsmark C.E., Tang G., Xu H., Tuft M., Hughes S.J., Yadav D. (2020). The Use of Pancreatic Enzyme Replacement Therapy in Patients with a Diagnosis of Chronic Pancreatitis and Pancreatic Cancer in the US is Infrequent and Inconsistent. Aliment. Pharmacol. Ther..

[65] Zdenkowski N., Radvan G., Pugliese L., Charlton J., Oldmeadow C., Fraser A., Bonaventura A. (2017). Treatment of Pancreatic Insufficiency Using Pancreatic Extract in Patients with Advanced Pancreatic Cancer: A Pilot Study (PICNIC). Support. Care Cancer.

[66] Roberts K., Bannister C., Schrem H. (2019). Enzyme Replacement Improves Survival among Patients with Pancreatic Cancer: Results of a Population Based Study. Pancreatology.

[67] Domínguez-Muñoz J.E., Nieto-Garcia L., López-Díaz J., Larińo-Noia J., Abdulkader I., Iglesias-Garcia J. (2018). Impact of the Treatment of Pancreatic Exocrine Insufficiency on Survival of Patients with Unresectable Pancreatic Cancer: A Retrospective Analysis. BMC Cancer.

[68] Yoshida T., Delafontaine P. (2015). Mechanisms of Cachexia in Chronic Disease States. Am. J. Med. Sci..

[69] Martin-Perez E., Domínguez-Muñoz J.E., Botella-Romero F., Cerezo L., Teresa F.M., Serrano T., Vera R. (2020). Multidisciplinary Consensus Statement on the Clinical Management of Patients with Pancreatic Cancer. Clin. Transl. Oncol..

[70] Sandini M., Patino M., Ferrone C.R., Alvarez-Pérez C.A., Honselmann K.C., Paiella S., Catania M., Riva L., Tedesco G., Casolino R. (2018). Association Between Changes in Body Composition and Neoadjuvant Treatment for Pancreatic Cancer. JAMA Surg..

[71] Naumann P., Eberlein J., Farnia B., Hackert T., Debus J., Combs S.E. (2019). Continued Weight Loss and Sarcopenia Predict Poor Outcomes in Locally Advanced Pancreatic Cancer Treated with Chemoradiation. Cancers.

[72] Cooper A., Slack R., Fogelman D., Holmes H.M., Petzel M., Parker N., Balachandran A., Garg N., Ngo-Huang A., Varadhachary G. (2015). Characterization of Anthropometric Changes that Occur During Neoadjuvant Therapy for Potentially Resectable Pancreatic Cancer. Ann. Surg. Oncol..

[73] Griffin O.M., Duggan S.N., Ryan R., McDermott R., Geoghegan J., Conlon K.C. (2019). Characterising the Impact of Body Composition Change During Neoadjuvant Chemotherapy for Pancreatic Cancer. Pancreatology.

[74] Probst P., Haller S., Bruckner T., Ulrich A., Strobel O., Hackert T., Diener M.K., Büchler M.W., Knebel P. (2017). Prospective Trial to Evaluate the Prognostic Value of Different Nutritional Assessment Scores in Pancreatic Surgery (NURIMAS Pancreas). Br. J. Surg..

[75] Cao Q., Xiong Y., Zhong Z., Ye Q. (2019). Computed Tomography-Assessed Sarcopenia Indexes Predict Major Complications Following Surgery for Hepatopancreatobiliary Malignancy: A Meta-Analysis. Ann. Nutr. Metab..

[76] Ratnayake B., Loveday B.P., Shrikhande S.V., Windsor J.A., Pandanaboyana S. (2018). Impact of Preoperative Sarcopenia on Postoperative Outcomes Following Pancreatic Resection: A Systematic Review and Meta-Analysis. Pancreatology.

[77] Trestini I., Paiella S., Sandini M., Sperduti I., Elio G., Pollini T., Melisi D., Auriemma A., Soldà C., Bonaiuto C. (2020). Prognostic Impact of Preoperative Nutritional Risk in Patients Who Undergo Surgery for Pancreatic Adenocarcinoma. Ann. Surg. Oncol..

[78] Peng P., Hyder O., Firoozmand A., Kneuertz P., Schulick R.D., Huang D., Makary M., Hirose K., Edil B., Choti M.A. (2012). Impact of Sarcopenia on Outcomes Following Resection of Pancreatic Adenocarcinoma. J. Gastrointest. Surg..

[79] Okumura S., Kaido T., Hamaguchi Y., Fujimoto Y., Masui T., Mizumoto M., Hammad A., Mori A., Takaori K., Uemoto S. (2015). Impact of Preoperative Quality as Well as Quantity of Skeletal Muscle on Survival after Resection of Pancreatic Cancer. Surgery.

[80] Bundred J., Kamarajah S.K., Roberts K.J. (2019). Body Composition Assessment and Sarcopenia in Patients with Pancreatic Cancer: A Systematic Review and Meta-Analysis. HPB.

[81] Mintziras I., Miligkos M., Wächter S., Manoharan J., Maurer E., Bartsch D.K. (2018). Sarcopenia and Sarcopenic Obesity are Significantly Associated with Poorer Overall Survival in Patients with Pancreatic Cancer: Systematic Review and Meta-Analysis. Int. J. Surg..

[82] Klute K.A., Brouwer J., Jhawer M., Sachs H., Gangadin A., Ocean A., Popa E., Dai T., Wu G., Christos P. (2016). Chemotherapy Dose Intensity Predicted by Baseline Nutrition Assessment in Gastrointestinal Malignancies: A Multicentre Analysis. Eur. J. Cancer.

[83] Basile D., Parnofiello A., Vitale M.G., Cortiula F., Gerratana L., Fanotto V., Lisanti C., Pelizzari G., Ongaro E., Bartoletti M. (2019). The IMPACT Study: Early Loss of Skeletal Muscle Mass in Advanced Pancreatic Cancer Patients. J. Cachexia Sarcopenia Muscle.

[84] Kurita Y., Kobayashi N., Tokuhisa M., Goto A., Kubota K., Endo I., Nakajima A., Ichikawa Y. (2019). Sarcopenia is a Reliable Prognostic Factor in Patients with Advanced Pancreatic Cancer Receiving FOLFIRINOX Chemotherapy. Pancreatology.

[85] Kays J.K., Shahda S., Stanley M., Bell T., O’Neill B.H., Kohli M.D., Couch M.E., Koniaris L.G., Zimmers T.A. (2018). Three Cachexia Phenotypes and the Impact of Fat-Only Loss on Survival in FOLFIRINOX Therapy for Pancreatic Cancer. J. Cachexia Sarcopenia Muscle.

[86] Lee H.S., Kim S.Y., Chung M.J., Park J.Y., Bang S., Park S.W., Song S.Y. (2019). Skeletal Muscle Mass Predicts Poor Prognosis in Patients with Advanced Pancreatic Cancer Undergoing Second-Line FOLFIRINOX Chemotherapy. Nutr. Cancer.

[87] Argiles J.M. (2005). Cancer-Associated Malnutrition. Eur. J. Oncol. Nurs..

[88] Caccialanza R., Lobascio F., Brugnatelli S., Pedrazzoli P. (2020). Nutritional Support in Pancreatic Cancer. Cancer.

[89] Arends J., Baracos V., Bertz H., Bozzetti F., Calder P.C., Deutz N.E., Erickson N., Laviano A., Lisanti M.P., Lobo D.N. (2017). ESPEN Expert Group Recommendations for Action Against Cancer-Related Malnutrition. Clin. Nutr..

[90] Tobert C.M., Mott S.L., Nepple K. (2018). Malnutrition Diagnosis during Adult Inpatient Hospitalizations: Analysis of a Multi-Institutional Collaborative Database of Academic Medical Centers. J. Acad. Nutr. Diet..

[91] Caccialanza R., De Lorenzo F., Pedrazzoli P. (2017). The Integrating Nutritional Therapy in Oncology (INTO) Project: Rationale, Structure and Preliminary Results. ESMO Open.

[92] Muscaritoli M., Arends J., Aapro M. (2019). from Guidelines to Clinical Practice: A Roadmap for Oncologists for Nutrition Therapy for Cancer Patients. Ther. Adv. Med. Oncol..

[93] Caccialanza R., Lobascio F., Cereda E., Aprile G., Farina G., Traclò F., Borioli V., Caraccia M., Turri A., De Lorenzo F. (2020). Cancer-Related Malnutrition Management: A Survey Among Italian Oncology Units and Patients’ Associations. Curr. Probl. Cancer.

[94] Caccialanza R., Pedrazzoli P., Cereda E., Gavazzi C., Pinto C., Paccagnella A., Beretta G.D., Nardi M., Laviano A., Zagonel V. (2016). Nutritional Support in Cancer Patients: A Position Paper from the Italian Society of Medical Oncology (AIOM) and the Italian Society of Artificial Nutrition and Metabolism (SINPE). J. Cancer.

[95] Thompson K.L., Elliott L., Fuchs-Tarlovsky V., Levin R.M., Voss A.C., Piemonte T. (2017). Oncology Evidence-Based Nutrition Practice Guideline for Adults. J. Acad. Nutr. Diet..

[96] Caccialanza R., Goldwasser F., Marschal O., Ottery F., Schiefke I., Tilleul P., Zalcman G., Pedrazzoli P. (2020). Unmet Needs in Clinical Nutrition in Oncology: A Multinational Analysis of Real-World Evidence. Ther. Adv. Med. Oncol..

[97] Rossi R., Serra P., Suzzi M., Guerra D., Bilotta S., Ricci M., Pallotti M.C., Ibrahim T., Frassineti G.L., Zavoiu V. (2020). The Challenge for Nutritional Care in a Cancer Center: The Need for Integration Between Clinical Nutritionist, Oncologist, and Palliative Care Physician. Curr. Probl. Cancer.

[98] Marín Caro M.M., Laviano A., Pichard C. (2007). Nutritional Intervention and Quality of Life in Adult Oncology Patients. Clin. Nutr..

[99] Hopkinson J.B. (2014). Psychosocial Impact of Cancer Cachexia. J. Cachexia Sarcopenia Muscle.

[100] Bayer L.M., Bauers C.M., Kapp S.R. (1983). Psychosocial Aspects of Nutritional Support. Nurs. Clin. N. Am..

[101] Ravasco P. (2019). Nutrition in Cancer Patients. J. Clin. Med..

[102] Muscaritoli M., Molfino A., Gioia G., Laviano A., Fanelli F.R. (2011). The Parallel Pathway: A Novel Nutritional and Metabolic Approach to Cancer Patients. Intern. Emerg. Med..

